# Reliability of estimating left ventricular ejection fraction in clinical routine: a validation study of the SWEDEHEART registry

**DOI:** 10.1007/s00392-022-02031-0

**Published:** 2022-05-17

**Authors:** Joel Lenell, Bertil Lindahl, Per Karlsson, Gorav Batra, David Erlinge, Tomas Jernberg, Jonas Spaak, Tomasz Baron

**Affiliations:** 1 Department of Medical Sciences, Cardiology, Uppsala Clinical Research Center, Uppsala University, Uppsala, Sweden; 2 Department of Cardiology and Clinical Physiology, Uppsala University Hospital, Uppsala, Sweden; 3 Department of Clinical Sciences, Cardiology, Lund University, Lund, Sweden; 4 Division of Cardiovascular Medicine, Department of Clinical Sciences, Karolinska Institute, Danderyd Hospital, Stockholm, Sweden

**Keywords:** LVEF, Registry, Validation, SWEDEHEART, Echocardiography

## Abstract

**Objective:**

Patients hospitalized with acute coronary syndrome (ACS) in Sweden routinely undergo an echocardiographic examination with assessment of left ventricular ejection fraction (LVEF). LVEF is a measurement widely used for outcome prediction and treatment guidance. The obtained LVEF is categorized as normal (> 50%) or mildly, moderately, or severely impaired (40–49, 30–39, and < 30%, respectively) and reported to the nationwide registry for ACS (SWEDEHEART). The purpose of this study was to determine the reliability of the reported LVEF values by validating them against an independent re-evaluation of LVEF.

**Methods:**

A random sample of 130 patients from three hospitals were included. LVEF re-evaluation was performed by two independent reviewers using the modified biplane Simpson method and their mean LVEF was compared to the LVEF reported to SWEDEHEART. Agreement between reported and re-evaluated LVEF was assessed using Gwet’s AC2 statistics.

**Results:**

Analysis showed good agreement between reported and re-evaluated LVEF (AC2: 0.76 [95% CI 0.69–0.84]). The LVEF re-evaluations were in agreement with the registry reported LVEF categorization in 86 (66.0%) of the cases. In 33 (25.4%) of the cases the SWEDEHEART-reported LVEF was lower than re-evaluated LVEF. The opposite relation was found in 11 (8.5%) of the cases (*p* < 0.005).

**Conclusion:**

Independent validation of SWEDEHEART-reported LVEF shows an overall good agreement with the re-evaluated LVEF. However, a tendency towards underestimation of LVEF was observed, with the largest discrepancy between re-evaluated LVEF and registry LVEF in subjects with subnormal LV-function in whom the reported assessment of LVEF should be interpreted more cautiously.

**Graphical abstract:**

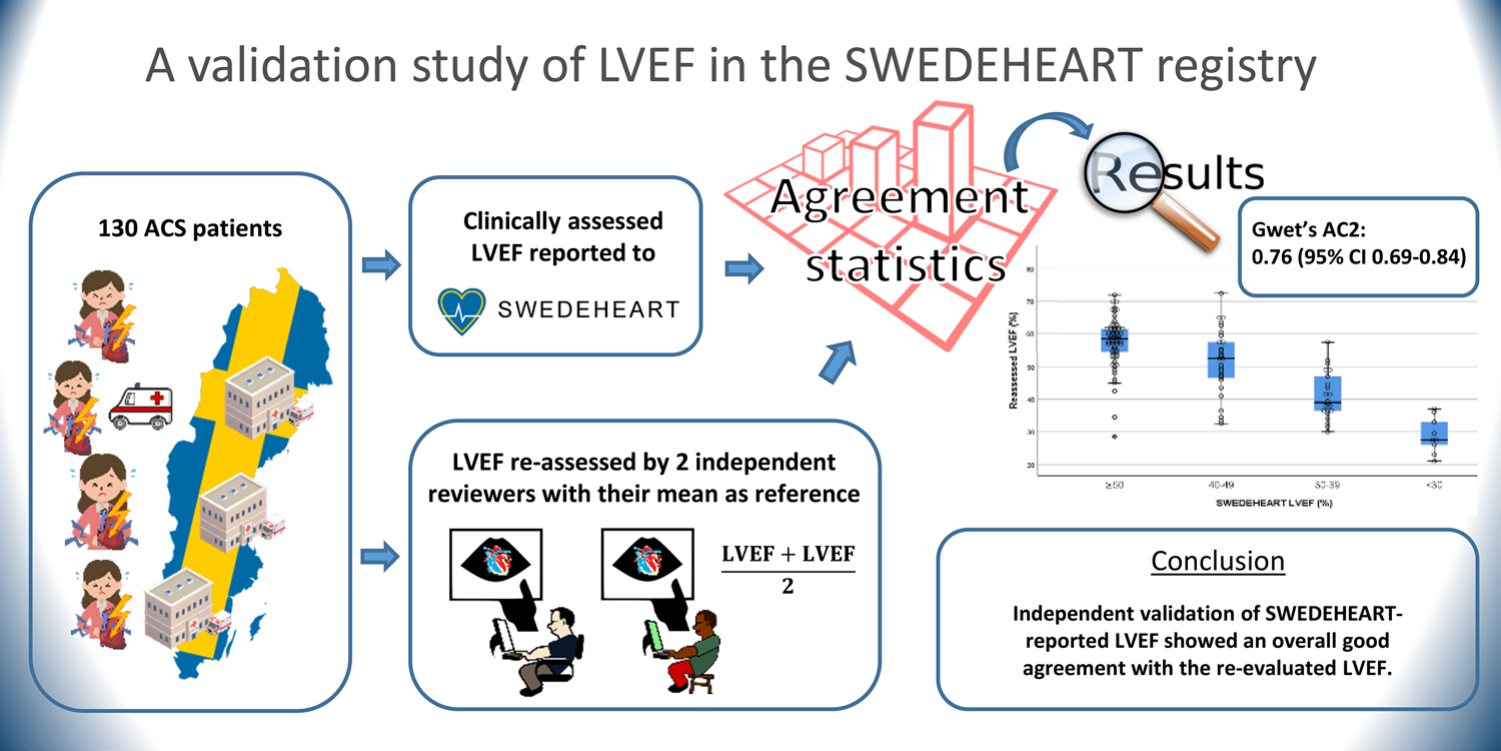

## Background

Left ventricular ejection fraction (LVEF) is one of the most robust predictive parameters post-myocardial infarction offering guidance in both treatment and follow-up strategies [1]. However, despite its recognized value, LVEF by biplane Simpson is subject to a non-negligible inter-observer variability that increases with declining image quality [2–4]. Poor image quality may prevent reliable delineation of the endocardial border and hinder a quantitative assessment of LVEF [5]. In such cases, LVEF may be assessed using visual “eye-balling”. This technique implicates an even greater uncertainty with a reported inter-observer variability of up to ± 14% LVEF [6].

The SWEDEHEART registry contains patient data from subjects admitted to Swedish hospitals nationwide due to acute coronary syndrome (ACS). The registry was established in 2009 by merging of four preexisting registries on ischemic heart disease into a larger, more comprehensive registry. The registry encompasses more than 450 variables including left ventricular ejection fraction (LVEF) by echocardiography [7]. SWEDEHEART has been a valuable source of insight in different aspects of ischemic heart disease and data from SWEDEHEART, including LVEF, has been used in a large number of studies [8].

Given the importance of LVEF and that the LVEF assessments in SWEDEHEART, and in several other registries, have not previously been validated we aimed to validate the LVEF assessments reported to SWEDEHEART.

## Methods

**Fig. 1:**
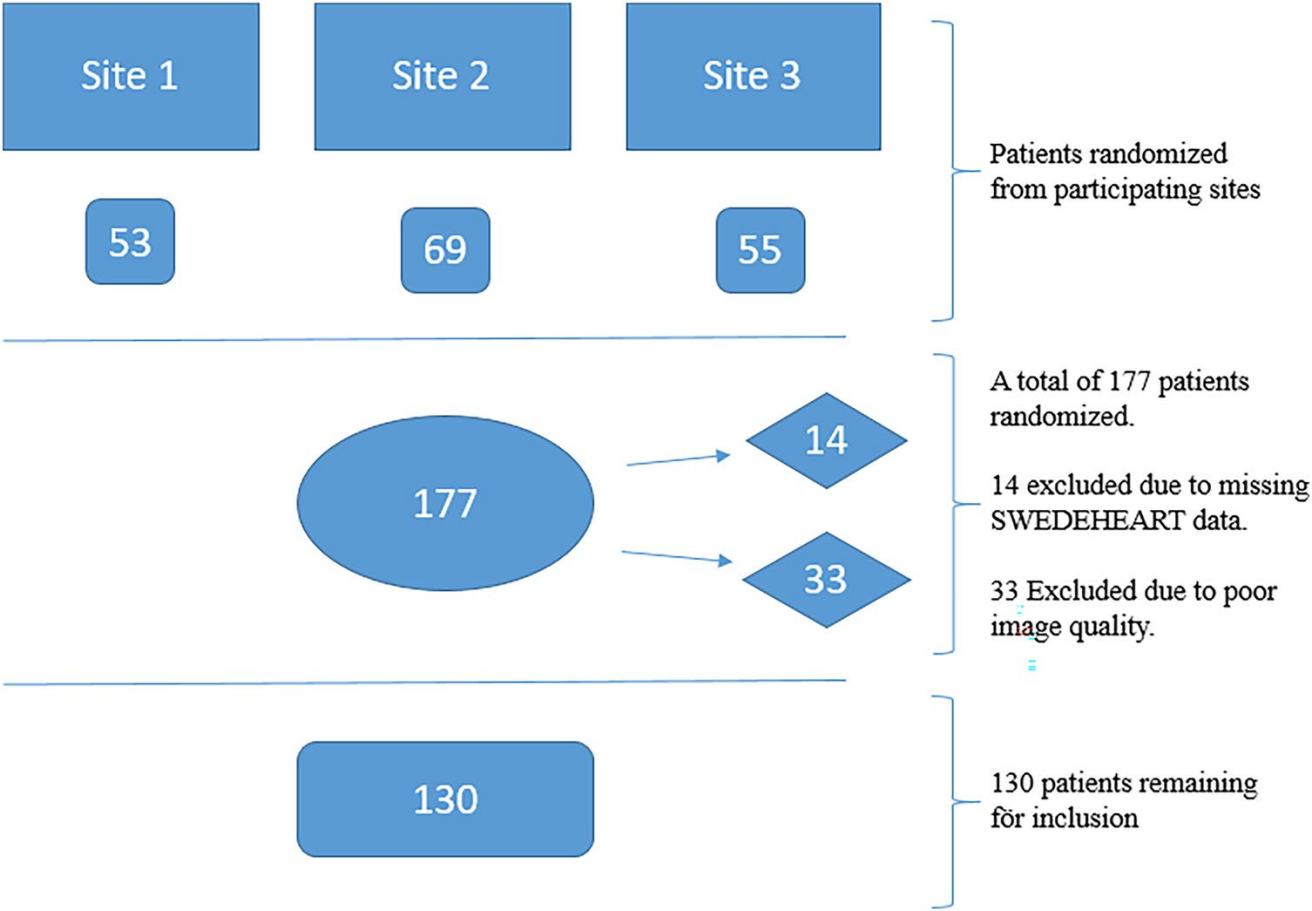
Flow chart illustrating patient selection

The study cohort consisted of a random sample of 177 patients with ACS from three different Swedish hospitals [Uppsala University Hospital, Uppsala (site 1), Lund University Hospital, Lund (site 2) and Danderyd University Hospital, Stockholm (site 3)], in whom LVEF had been assessed according to local routine by 2D echocardiography during the index hospital stay. LVEF was then reported to SWEDEHEART in four categories: < 30%, 30–39%, 40–49%, and ≥ 50%. Missing data on LVEF in the registry prompted exclusion from the study (Fig. 1).

The echocardiographic raw data was collected from the imaging databases at the participating hospitals and LVEF was re-evaluated at the core lab in Uppsala by two independent reviewers according to the modified biplane Simpson method using TOMTEC-ARENA TTA2 software (TOMTEC IMAGING SYSTEMS, GMBH EDISONSTRASSE, Unterschleissheim DE) with manual delineation of the endocardial border [9]. Subjects with more than two untraceable myocardial segments due to insufficient image quality were excluded. The reviewers were experienced echocardiographers blinded to all patient related clinical data. The mean LVEF value of the two reassessments was used as reference when compared to SWEDEHEART data. Before comparison, the reassessed reference values were categorized from continuous into ordinal scale variables in accordance with the LVEF ranges found in SWEDEHEART.

### Statistical analysis

Spearman’s rank correlation was used to test correlation and Wilcoxon signed-rank test was used to assess bias. Gwet’s AC1 and AC2 statistics were used to test agreement between SWEDEHEART data and the reassessments. The Gwet’s AC2 analysis was performed with pre-specified linear weights. The Intraclass correlation coefficient (ICC) was used for inter-observer variability assessment between the two reference reviewers by a two-way mixed-effects model examining consistency in “single rater” type [10, 11]. The analysis was performed using SPSS software 26.0, SPSS Inc., Chicago, IL, USA and R (4.0.2) with statistical significance defined by *p* < 0.05. The results were presented in a tabular format with 95% confidence intervals. The study was approved by the Regional Committee for Medical Research Ethics (DNR 2017/759-31).

## Results

The median age in the study population was 65 years, and 76% were men. See Table 1 for further baseline characteristics.

**Table 1:** Patient demographics and medical history

Characteristics	*n* = 130
Age, years median (IQR)	65 (58–72)
Male sex (%)	99 (76)
Active smoker, (%)	36 (28)
Diagnosis of ACS	
STEMI (%)	65 (50)
NSTEMI (%)	62 (48)
Other (UA, type 2 MI, Takotsubo) (%)	3 (2)
Medical history	
Hypertension (%)	72 (55)
Diabetes mellitus (%)	27 (21)
Atrial fibrillation (%)	6 (5)
Heart failure (%)	22 (17)
History of stroke (%)	10 (8)
History of MI (%)	29 (22)

*IQR* Inter quartile range, *STEMI* ST-elevation myocardial infarction, *NSTEMI* non-ST-elevation myocardial infarction, *BMI* Body mass index, *UA* Unstable angina pectoris

**Fig. 2:**
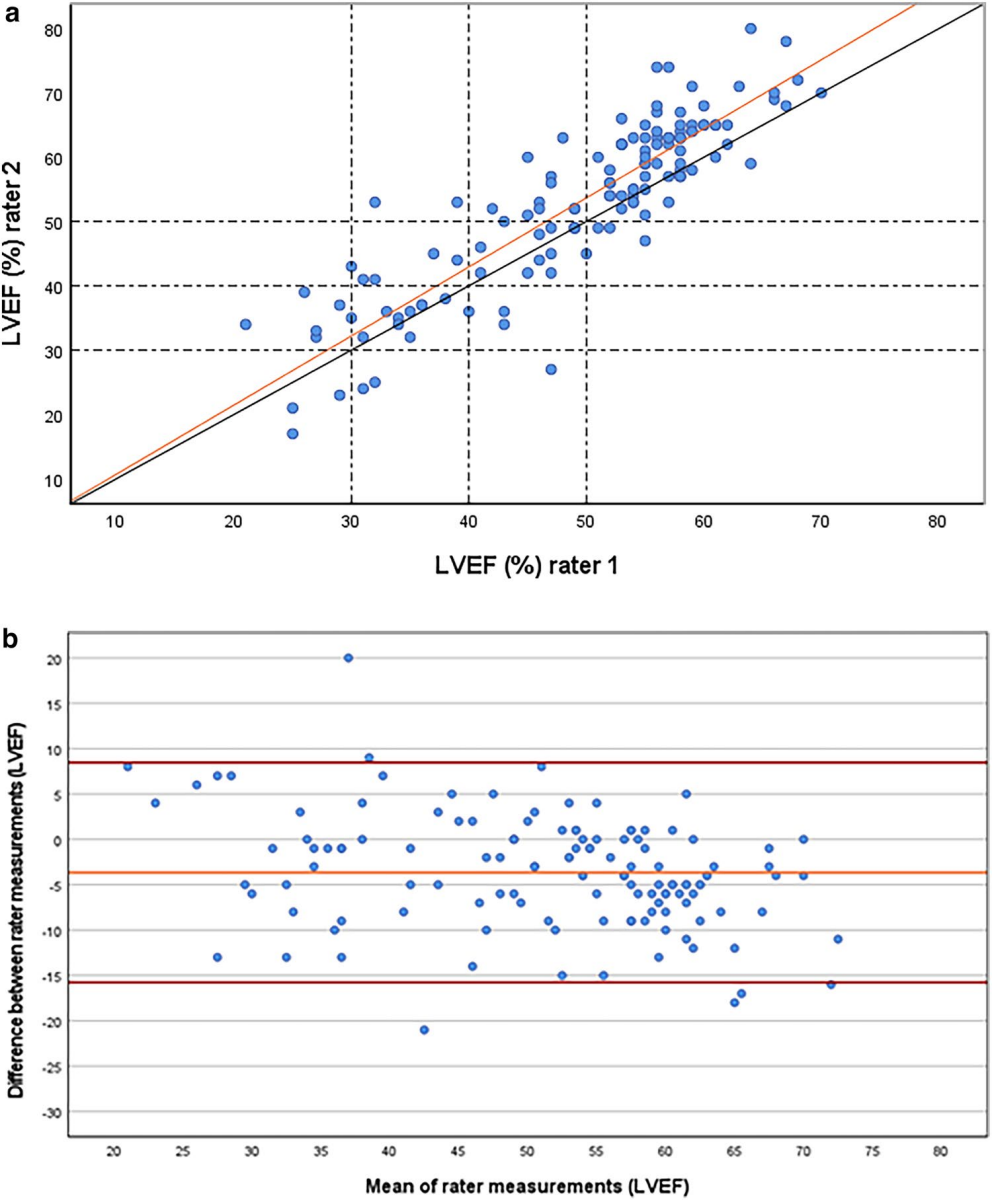
**a** A scatter plot illustrating inter-observer variability between the two reviewers. The dotted lines mark the cut-off values defining LVEF categories in SWEDEHEART. **b** Bland–Altman plot illustrating the difference between the reviewers’ measurements to their mean. Orange line = mean difference, Red lines = mean difference ± 1.96* standard deviation of the difference

After LVEF was re-evaluated by the two reviewers with the modified biplane Simpson method, the mean LVEF was calculated for comparison with the SWEDEHEART data. The inter-observer variability between the two reference reviewers re-evaluating LVEF by biplane Simpson was close to excellent [ICC 0.87 (95% CI 0.82–0.92)]. Likewise, there was an excellent correlation with Spearman’s *R* of 0.88 (*p* < 0.001). Figure 2a displays a scatterplot of the reassessments by the two reviewers and Fig. 2b illustrates their agreement in a Bland–Altman plot.

**Fig. 3:**
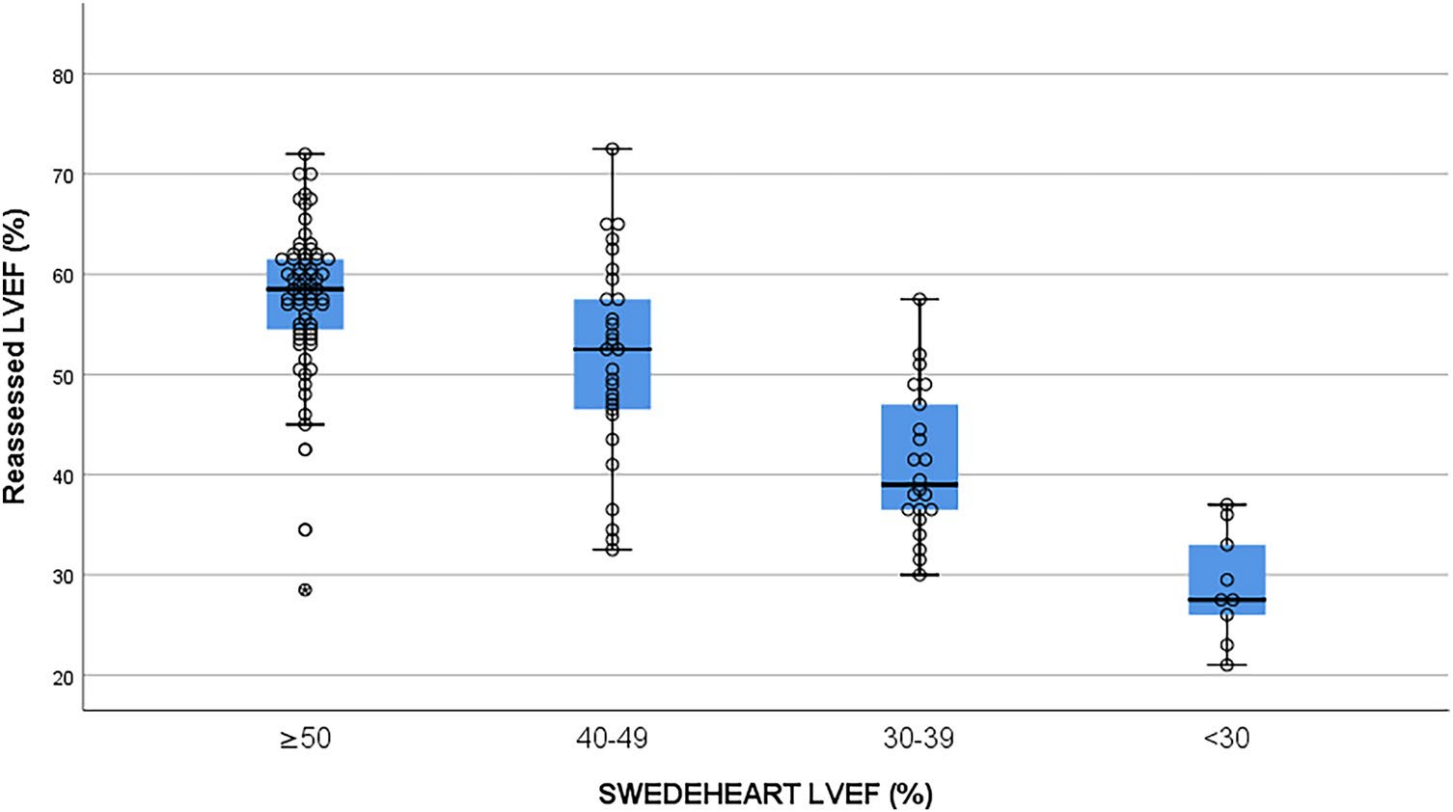
Combined boxplot and dotplot with distribution of the reassessed LVEF in LVEF categories according to SWEDEHEART. Middle line = median; Box = interquartile range (IQR); Whiskers = lowest and maximum LVEF excluding outliers; Circle outside whiskers = mild outliers; asterisk in circle = severe outliers

As displayed in Table 2 there is an overall good correlation between reassessed LVEF and SWEDEHEART LVEF (Spearman’s *r* = 0.69, *p* < 0.01). Agreement as assessed by Gwet’s AC1 was moderate [0.58 (95% CI 0.47–0.69)], however, when adjusted for misclassification errors (AC2), the agreement between SWEDEHEART LVEF and the gold standard was good [0.76 (95% CI 0.69–0.84)]. There was a trend towards lower LVEF values in the registry when compared to the reassessments. This bias was further explored in Table 3 by the Wilcoxon signed-rank test. The analysis showed a lower category LVEF in 25.4% (*p* < 0.001) of cases in SWEDEHEART and an absolute agreement in 66% of cases. Figure 3 displays a particularly wide distribution of reassessed LVEF among patients registered in the LVEF range between 40 and 49%, with a median above the 50% cut-off limit. There appears to be a slight bias towards registering a lower category LVEF in subjects with borderline function.

**Table 2:** Crosstab displaying the overlap and distribution of the categorized re-evaluated LVEF (gold standard) and the LVEF registered in SWEDEHEART

SWEDEHEART	Reassessed LVEF				
	LVEF ≥ 50%	LVEF 40–49%	LVEF 30–39%	LVEF < 30%	Total
LVEF ≥ 50%	62	5	1	1	69
LVEF 40–49%	18	8	4	0	30
LVEF 30–39%	3	8	11	0	22
LVEF < 30%	0	0	4	5	9
Total	83	21	20	6	130

**Table 3:** A Wilcoxon signed-rank test of bias between re-evaluated LVEF and LVEF estimates in the SWEDEHEART registry

Wilcoxon signed-rank test	*n* (%)
Reassessed LVEF > SWEDEHEART	33 (25.4)
Reassessed LVEF < SWEDEHEART	11 (8.6%)
Ties	86 (66.0)
Total	130

## Discussion

Independent validation of LVEF in the SWEDEHEART registry shows an overall good agreement when re-evaluated by two independent reviewers using the modified biplane Simpson method. However, there was a tendency towards underestimation of LVEF in SWEDEHEART, with the largest discrepancy between re-evaluated LVEF and SWEDEHEART LVEF in subjects with LVEF < 50%. Only five patients had a greater than one category difference.

To our knowledge this is the first validation study of LVEF assessed by echocardiography in a quality registry by reassessment of the raw data. Consequently, it may be difficult to put these results in relation to previous registry validations. In 2016, Govatsmark et al. published a validation of the Norwegian myocardial infarction register with medical records as reference, without reinterpretation of the echocardiography raw data [12]. This approach, with medical records as gold standard, is common in registry validation studies, however, it fails to address potential flaws in index data acquisition [13, 14]. Publications on inter-observer variability in LVEF may be more feasible as a benchmark since they more closely reflect this SWEDEHEART validation study design. Such studies, covering different clinical settings, have presented good to excellent inter-observer agreement of LVEF in populations with preserved ejection fraction, yet only moderate agreement in subjects with subnormal ejection fraction [15–17]. The findings from the SWEDEHEART registry are thus in accordance with previous results.

There were 47 subjects (36%) with LVEF < 50% in this study sample collected between 2008 and 2014. As of 2014, 35% of all the subjects registered to SWEDEHEART had an LVEF < 50%, indicating that the study sample was representative of the overall population [18].

Assessment of LVEF by visual eye-balling was, at the time of enrollment, the most common method of LVEF assessment at all three participating centers. This approach has repeatedly shown to underestimate LVEF compared to quantitative assessment by the modified Simpson method [19–21]. Visually assessed LVEF has substantial inter-observer variability of up to ± 14% LVEF [6]. In such settings, grouping the values into larger categories might be reasonable. However, current guidelines on cardiac chamber quantification favor the more robust modified biplane Simpson approach that generates a continuous variable [22]. In such quantitative setting, it may be more reasonable to report the obtained LVEF value to avoid the obvious loss of granularity in categorized data. Categorization may also increase the perceived difference between two assessments that end up on different sides of a cut-off limit, despite being close in numeric value. The current categorization also fails to harmonize with important clinical cut-off limits such as the 35% LVEF limit in deciding on ICD and CRT implantation [23]. Algorithms enabling an automatic tracing of the endocardial border, generating an instant assessment of LVEF, are now common and validated tools in the echocardiography lab [24]. These techniques further lower the barriers to a quantitative assessment of a continuous LVEF variable. However, should quantitatively assessed LVEF come to be reported as a continuous variable it must still be interpreted with respect to a smaller, yet significant, inter-observer variation and a smallest detectable change of almost 7 % points [3, 25].

Kappa statistics has repeatedly been used in inter-observer variability studies on LVEF due to its property of adjusting for chance agreement [26]. However, Cohen’s kappa is prone to underestimating agreement in populations with symmetrical imbalance [26] [27]. As the majority (63%) of the studied SWEDEHEART subjects presented with a preserved ejection fraction (LVEF > 50%), Cohen’s kappa was rendered unfeasible in this setting. Instead, the weighed Gwet’s AC2 method offered a more robust analysis, yet despite its advantages, it appears to be rarely performed in publications on assessments of LVEF agreement [12, 28, 29].

There was an agreement on categorized LVEF in roughly 50% of the cases with reduced LVEF (as determined by the reference method). This discrepancy is largest in the mid-range categories of 30–49% where important clinical cut-off limits are present. An improved precision of LVEF assessments in this subgroup would be most favorable. A Japanese study examined two different teaching interventions with regard to improved inter-rater variability in visually assessed LVEF [30]. The interventions significantly reduced the misclassification rates of LVEF regardless of operator experience and image quality. There are several other studies in support of similar teaching interventions [6, 31]. Such initiatives could possibly further improve the quality of LVEF assessments entered in SWEDEHEART.

### Limitations

There is a theoretical risk that some LVEF assessments have been misclassified into the wrong category in SWEDEHEART since this process is performed manually. As we have not validated the registry LVEF in relation to patient records such faults cannot be accounted for in this study which may be considered a limitation. The extreme outlier in Fig. 3 may be an example of such accidental misclassification. LVEF lacks a gold standard and, as a consequence, the proper reference method in LVEF validation studies is a subject open for debate. Given that alternative imaging modalities, such as cardiac magnetic resonance imaging (CMR) or cardiac computed tomography (CCT), have not been routinely performed in Swedish ACS subjects we deemed it appropriate that the reference in this study should be based on the 2D echocardiographic raw data. The use of a mean from two independent reassessments by the modified Simpson rule was proposed to strengthen the robustness in the reference method, however, there is scarce literature in support of this notion.

The patients were included at three university hospitals which may give rise to questions of sample representation since SWEDEHEART contains data from all Swedish hospitals treating ACS. A larger nationwide follow-up with a greater variation in hospital size may provide additional information on the data validity in SWEDEHEART. However, as previously mentioned, the representability of the material is supported by the concurring proportion of subjects categorized with reduced ejection fraction in this study and in the SWEDEHEART registry [18].

## Conclusion

Independent validation of SWEDEHEART-reported LVEF shows an overall good agreement with the re-evaluated LVEF. However, a tendency towards underestimation of LVEF was observed, with the largest discrepancy between re-evaluated LVEF and registry LVEF found in subjects with subnormal LV-function in whom the reported assessment of LVEF should be interpreted more cautiously.

## Data Availability

The data may be provided upon request.
